# Aggression supersedes individual oxygen demand to drive group air‐breathing in a social catfish

**DOI:** 10.1111/1365-2656.12758

**Published:** 2017-10-30

**Authors:** Shaun S. Killen, Andrew J. Esbaugh, Nicolas F. Martins, F. Tadeu Rantin, David J. McKenzie

**Affiliations:** ^1^ Institute of Biodiversity, Animal Health and Comparative Medicine College of Medical, Veterinary and Life Sciences University of Glasgow Glasgow UK; ^2^ Department of Marine Science Marine Science Institute University of Texas at Austin Port Aransas TX USA; ^3^ Department of Physiological Sciences Federal University of São Carlos São Carlos Brazil; ^4^ Centre for Marine Biodiversity Exploitation and Conservation UMR9190 (IRD, Ifremer, UM, CNRS) Université Montpellier Montpellier Cedex 5 France

**Keywords:** air‐breathing fish, ecophysiology, group‐living, keystone individuals, metabolic rate, social behaviour

## Abstract

1. Group‐living is widespread among animals and comes with numerous costs and benefits. To date, research examining group‐living has focused on trade‐offs surrounding foraging, while other forms of resource acquisition have been largely overlooked.
2. Air‐breathing has evolved in many fish lineages, allowing animals to obtain oxygen in hypoxic aquatic environments. Breathing air increases the threat of predation, so some species perform group air‐breathing, to reduce individual risk. Within species, individual air‐breathing can be influenced by metabolic rate as well as personality, but the mechanisms of group air‐breathing remain unexplored. It is conceivable that keystone individuals with high metabolic demand or intrinsic tendency to breathe air may drive social breathing, especially in hypoxia.
3. We examined social air‐breathing in African sharptooth catfish *Clarias gariepinus*, to determine whether individual physiological traits and spontaneous tendency to breathe air influence the behaviour of entire groups, and whether such influences vary in relation to aquatic oxygen availability.
4. We studied 11 groups of four catfish in a laboratory arena and recorded air‐breathing behaviour, activity and agonistic interactions at varying levels of hypoxia. Bimodal respirometry was used to estimate individual standard metabolic rate (SMR) and the tendency to utilize aerial oxygen when alone.
5. Fish took more air breaths in groups as compared to when they were alone, regardless of water oxygen content, and displayed temporally clustered air‐breathing behaviour, consistent with existing definitions of synchronous air‐breathing. However, groups displayed tremendous variability in surfacing behaviour. Aggression by dominant individuals within groups was the main factor influencing air‐breathing of the entire group. There was no association between individual SMR, or the tendency to obtain oxygen from air when in isolation, and group air‐breathing.
6. For *C. gariepinus*, synchronous air‐breathing is strongly influenced by agonistic interactions, which may expose subordinate individuals to risk of predation. Influential individuals exerted an overriding effect on risk‐taking by the entire group, for reasons independent of their physiological oxygen requirements. Overall, this illustrates that social context can obscure interactions between an individual's physiological and behavioural traits and their tendency to take risks to obtain resources.

## 1 INTRODUCTION

Group‐living is ubiquitous throughout the animal Kingdom and comes with a variety of costs and benefits (Krause & Ruxton, 2002; Ward & Webster, 2016). For example, individuals in groups experience reduced predation risk via a variety of mechanisms (Krause & Ruxton, 2002; Pitcher & Parrish, 1993; Ward & Webster, 2016) and also have increased foraging efficiency (Ekman & Hake, 1988; Pitcher, Magurran, & Winfield, 1982; Ruxton, Hall, & Gurney, 1995). However, living within groups can also increase intraspecific aggression and competition for food (Killen, Fu, Wu, Wang, & Fu, 2016; Webster & Hart, 2006). To date, the costs and benefits of group membership have almost exclusively focused on the trade‐off between foraging and predation risk, while other forms of resource acquisition remain largely unstudied (McKenzie, Belão, Killen, & Rantin, 2015).

Oxygen is another essential resource that can be difficult or risky for some animals to obtain. This is especially true for fishes because water contains much less oxygen than air and requires more effort for ventilation. An ability to breathe air has evolved independently in numerous lineages of bony fish, in at least 370 species from 49 families, underscoring the potential advantages of being able to access this rich source of oxygen (Graham, 1994, 1997; Randall, Burggren, Farrell, & Haswell, 1981). Among freshwater fishes, an ability to breathe air is most common in tropical species from habitats that regularly experience aquatic hypoxia (Graham, 1997; Randall et al., 1981). All air‐breathing fishes are bimodal breathers meaning that, although they have an air‐breathing organ, they still possess gills to breathe water. Air‐breathing has a clear physiological drive; it is a reflex response driven by oxygen chemoreceptors that monitor water and blood oxygen levels. Within air‐breathing species, there is considerable variation among individuals in their reliance on aerial respiration, which is dependent upon their metabolic rate and oxygen demand (Lefevre, Wang et al., 2014; McKenzie, Burlesson, & Randall, 1991; McKenzie et al., 2015; Smatresk, Burleson, & Azizi, 1986). In addition, although it is a chemoreflex, air‐breathing in fishes also has a large behavioural component (Chapman & McKenzie, 2009; Video S1). To perform an air breath, the fish approaches and rapidly breaches the surface of the water with its head or mouth and quickly “gulps” air before retreating to depth (Video S1; Domenici et al., 2015; Graham, 2006). Although this behaviour can allow individuals to access aerial oxygen, it is inherently risky because the individual goes from being invisible to aerial or terrestrial predators, while in deep or murky water, to being visible at or near the water surface. Indeed, fishes with bimodal breathing can experience disproportionately high amounts of predation mortality, particularly under hypoxic conditions when they are driven to breathe air (Kramer, Manley, & Bourgeois, 1983). Among individuals, there is variation in the willingness to breath air when the behaviour might be risky, which is related to individual boldness (McKenzie et al., 2015).

To offset the risk associated with surfacing, many air‐breathing species engage in synchronous air‐breathing, whereby several individuals surface simultaneously or within a short time period (Chapman & Chapman, 1994; Kramer & Graham, 1976). This social surfacing may reduce risk of predation for any given individual (Kramer & Graham, 1976), in a manner similar to group foraging in fish schools or other animal groups. The cues involved in coordinating social air‐breathing are not understood, nor are the physiological factors that may influence this group behaviour. Given that individual fish differ in their need or willingness to breathe air when in isolation (McKenzie et al., 2015), the occurrence of synchronous air‐breathing implies that at least some individuals may be making a compromise to conform with the rest of the group by air‐breathing either more or less than, ideally, they would prefer (Webster & Ward, 2011). In particular, it is plausible that certain individuals may influence the behaviour of the entire group, namely those with a higher metabolic oxygen demand, those more willing to take risks to obtain the resource or those that are less attuned to risk. The strength of these influences may also be modulated by the degree of aquatic hypoxia.

Thus, group air‐breathing in fishes provides a useful model to examine the interplay between individual variation in physiological traits and the behaviour of entire groups, and how this is affected by the need to obtain a resource. It has recently been proposed that keystone individuals can have a disproportionate effect on the behaviour and success of all other individuals within social groups (Modlmeier, Keiser, Watters, Sih, & Pruitt, 2014; Pruitt & Keiser, 2014). Synchronous air‐breathing in fishes may provide novel insights into this role of individuals in guiding group behaviours in animals, particularly the role of physiological traits. We therefore studied social air‐breathing in groups of African sharptooth catfish *Clarias gariepinus*, a species that displays wide individual variation in air‐breathing activity (McKenzie et al., 2015). It is a social species that forms dominance hierarchies in the wild. We combined observations of air‐breathing in groups with estimates of individual metabolic rate and reliance on air‐breathing when alone, to investigate mechanisms driving social air‐breathing. Specifically, we aimed to address the following questions: (1) Does individual variation in metabolic rate or spontaneous tendency to breathe air affect group surfacing behaviours; and (2) Do key individuals influence group air‐breathing, and if so, does this influence change with environmental oxygen availability? The results provide insights into the drivers of group behaviour and the role of individual variation in the functioning of animal collectives.

## 2 MATERIALS AND METHODS

### 2.1 Animals

Juvenile *C. gariepinus* of unknown sex (mean mass ± *SD* = 64.1 ± 11.34 g; total length = 220 ± 10.85 mm) were obtained from Piscicultura Polettini (Mogi Mirim, SP, Brazil) and transported by road to the Department of Physiological Sciences, Federal University of São Carlos (São Carlos, SP). There, they were maintained in tanks supplied with well water at 25 ± 1°C under an approximate 12D:12L natural photoperiod and fed commercial feed at about 2% body mass per day, for 2 weeks. Animals were then tagged for a permanent method of identification throughout the study using a passive integrated transponder (BTS‐ID; bts‐id.com) into the dorsal epaxial muscle under mild anaesthesia (0.1 g/L benzocaine), after which they recovered in routine holding conditions for at least 72 hr before experiments.

### 2.2 Bimodal respirometry

Catfish were fasted for 24 hr prior to measurements, and then, individuals were transferred gently to bimodal respirometers (Lefevre, Bayley, & McKenzie, 2016; McKenzie et al., 2015). Four respirometers were partially immersed in two baths of well‐aerated water (0.4 m^2^ surface area, 12 cm water depth) with each setup partially screened behind opaque black plastic sheeting so that routine air‐breathing behaviours were not inhibited by fear of human presence (Lefevre, Wang, Phuong, & Bayley, 2011; Shingles, McKenzie, Claireaux, & Domenici, 2005). Water was maintained at 26.5 ± 0.5°C using submersible aquarium heaters. Fish were placed in the respirometers in the evening between 18.00 and 19.00 hr for respirometry data collected with the animals undisturbed for the ensuing 23 hr.

The bimodal respirometers had a total volume of 2.5 L, comprising an underwater phase and an air phase that projected above the water surface. The air phase had a volume of ~200 ml, and for each respirometer, the exact volume of the air phase was derived from the effects on O_2_ partial pressure of boluses of 100% N_2_ delivered into the sealed space (Lefevre et al., 2016). The remainder of the 2.5 L was the water phase volume. The relative amount of total O_2_ uptake that was met by water vs. air‐breathing was measured with an intermittent stopped flow technique (Steffensen, 1989) modified for bimodal respirometry (Lefevre et al., 2016; McKenzie et al., 2015). Briefly, the method alternated two periods within a 15‐min cycle. In the first, water and air chambers were closed to the exterior for 10 min, so that O_2_ uptake from both phases could be recorded. The alternate period was when the two phases, aquatic and aerial, were flushed simultaneously for 5 min to replenish O_2_ levels.

Oxygen levels in the water and air were measured with two PC‐controlled fibre‐optic oxygen meters with four channels each (Firesting, www.pyro-science.com), such that one metre measured data for one pair of respirometers. Optodes were positioned to sample water and air for each respirometer (Lefevre et al., 2016), with all data stored on a PC using the manufacturer's software. Absolute rates of O_2_ uptake from air (MO_2AIR_) and water (MO_2WATER_) were calculated (in mmol O_2_ kg^−1^ hr^−1^) for each 15‐min respirometry cycle, based upon the decline in O_2_ content in each phase during the closed period, as described previously (McKenzie et al., 2007; McKenzie, Steffensen, Taylor, & Abe, 2012). Data were collected starting from 6 hr after the fish were placed in the respirometers, hence from about 00:30 until they were removed at about 17:30, an interval of 17 hr. The MO_2AIR_ and MO_2WATER_ were calculated for this time interval for each individual and summed to calculate routine metabolic rate (RMR). The proportion of RMR derived from air‐breathing (%MO_2AIR_) was calculated as the percentage of total oxygen uptake over the measurement interval, for each individual, and was used in subsequent analyses. The standard metabolic rate (SMR) of each individual was estimated by the quantile method (Dupont‐Prinet et al., 2010; Chabot, Steffensen, & Farrell, 2016). This assumes that a certain proportion of the measures of RMR are below true SMR because of temporal variability and possible measurement errors (Chabot et al., 2016). The quantile splits the dataset into the *q* smallest and the 1−*q* largest values, where *q* is a proportion chosen by the experimenter. All measures of RMR were considered for each individual, hence 68 measures over 17 hr, and *q* was fixed at 0.12 such that 12% of values fell below true SMR (McKenzie et al., 2015; Chabot et al., 2016).

### 2.3 Behavioural observations

#### 2.3.1 Series I: group observations

Immediately following measurements of oxygen uptake, the four fish were moved to a behavioural arena for measurements of group air‐breathing behaviour. This was done on 11 cohorts of fish, overall providing data on individual oxygen uptake and group behaviour for 44 fish, split into 11 groups of four fish each. Group observations were performed in a 250‐L circular tank equipped with a 25‐L overflow reservoir. A recirculation pump was placed in the overflow reservoir, which returned water to the primary observation tank. Aeration was performed in the overflow tank to prevent any disturbance to the animals and video quality. Oxygen levels were monitored using a galvanic oxygen electrode (OxyGuard mini probe, www.oxyguard.dk) placed in‐line from the recirculation reservoir. The main observation tank was lined with white waterproof fabric to enhance contrast and eliminate reflection. Three lamps were mounted on the edge of the observation tank to enhance visibility through indirect illumination. Although this arena was brighter than what the fish would experience in a more natural setting, the fish appeared to behave normally. In addition, the open conditions in the arena served to heighten the potential trade‐off between air‐breathing and exposure to perceived risk. Behavioural observations were taken using a remotely controlled GoPro camera (gopro.com) mounted directly above the observation tank.

Group behaviour was monitored following respirometry analysis. Fish were anesthetized in a benzocaine solution (100 mg/L), and a coloured bead was sutured to their dorsal side. This provided a temporary means of visual identification during automated tracking of behaviour from video recorded during the trials. Fish were allowed to recover in isolation for 30–60 min after which they were placed in the observation tank and allowed to acclimate overnight (15 hr). The tank was flushed with clean water for 45 min the following morning, and the fish were allowed to further acclimate for 15 min post‐flushing. Video was collected for 15 min at five oxygen levels (100%, 80%, 60%, 40% and 20%) followed by recovery to 100%. Oxygen was lowered by bubbling 100% nitrogen into the overflow reservoir, and hypoxia levels were controlled by a feedback solenoid system (OXY‐REG, Loligo Systems, Denmark) connected to the oxygen electrode (OxyGuard A/S, Denmark). Preliminary experiments determined that 30–40 min of equilibration time was required to lower the observation tank to the required oxygen level, while 90 min of aeration was required to return the observation tank to 100%. Following final observations, fish were returned to the holding tanks and water was fully exchanged.

#### 2.3.2 Series II: individual observations

Individual behavioural observations were performed approximately 3 days following the group trials for group of fish. These observations were performed in a flow through 100‐L tank consisting of an inflow header chamber and a secondary aeration chamber that drained equally into six isolated observation chambers. Flow was controlled by a gravity overflow reservoir, which drained into the inflow header chamber. Oxygen levels were monitored using a PreSens Oxy‐4 mini fibre‐optic system and associated software (www.pre-sens.com). One electrode was placed in the aeration chamber, and a second was placed in the outflow from the observation chambers. The entire system was shielded from visual disturbance using a white sheet, and two lamps were mounted to the tank to provide indirect illumination. Fish were identified, placed in the individual observation chambers and allowed to acclimate overnight (15 hr). A cage lid was put in place to prevent animals from jumping out of the tank. The following morning the lid was removed and two GoPro cameras were put in place, after which the animals were acclimated for an additional 15 min prior to behavioural observations. Normoxic observations were performed for 15 min after which the lid was put back in place and oxygen levels were reduced by bubbling nitrogen into the aeration chamber. Oxygen levels were reduced to approximately 20% in 60–90 min (approximately the same duration as in the group trials in series 1) and subsequently maintained using a cyclical timer controlled solenoid. Once the desired oxygen level was reached, the lid was removed and fish were allowed to acclimate for 15 min prior to video collection. After final observations, fish were returned to the holding tanks.

### 2.4 Video analysis

Video recordings were analysed, and the behaviour of each individual within each group and at each level of oxygen availability was recorded using Solomon Coder (v. 14.05.18; Budapest, Hungary). For each fish, we quantified four behaviours: (1) breaths, defined as anytime the fish broke the surface of the water, opened its mouth and ingested air; (Video S1) (2) attacks, when a fish would bite or quickly lunge at a conspecific; (3) pushes, when a fish would displace another individual by nudging or steadily pushing it; and (4) avoids, when a fish would actively retreat from an attack or a push performed by a conspecific. The total distance moved by each individual at each level of oxygen availability was measured using Ethovision XT 10 (Noldus). For isolated trials, videos were analysed to quantify the number of breaths taken by each fish, again using Solomon Coder (v. 14.05.18; Budapest, Hungary).

### 2.5 Statistical analysis

All analyses were conducted using r v. 3.4.0 (R Development Core Team, 2017) using the function lmer in package lme4 (Bates et al., 2016) and MuMIn 1.9.13 (Barton, 2015) (http://CRAN.R-project.org/package=MuMIn). The effect of social context (isolation vs. group) on air‐breathing frequency was assessed using a linear mixed effects model (LME) with breaths as the dependent variable, social treatment and oxygen availability (normoxia and 20% air saturation) as fixed factors, and fish ID as a random factor. Individual variation in behaviour while in groups was further analysed using LMEs with breaths as the dependent variable, and log mass, log SMR, %MO_2AIR_, activity (log‐transformed total distance moved), attacks (log‐transformed) and oxygen concentration (categorical variable) as explanatory variables, along with all possible interactions with oxygen concentration. Fish ID nested with group was included as a random effect in this model. Additional models examined the factors influencing either log attacks or log activity as the dependent variables, and log mass, log SMR, %MO_2AIR_ and oxygen concentration (categorical variable) as explanatory variables. Again, fish ID nested within group was included as a random effect in these models. In all cases, the full model was first fitted using restricted maximum likelihood estimation (REML) to compare possible random structures by likelihood ratio testing. We compared the random intercept model with fish identity nested within group as a random factor with random slope models where the slope estimates were allowed to vary among individuals for oxygen availability. In all cases, random slopes did not improve model parsimony and so only random intercepts were used for fish ID nested within group. Temporal clustering (i.e. the degree of synchronization) of breaths and attacks when in groups was assessed by calculating the coefficient of dispersion (CD) for each 15‐min trial (Chapman & Chapman, 1994). Each observation was split into 30‐s intervals, to help account for “chains” of clustered surfacing behaviour occurring over tens of seconds but with only several seconds between surfacing events by different fish. The CD was then calculated as the variance/mean ratio across intervals: values greater than one indicate temporally clumped distributions, while values less than one indicate more uniform distributions. Changes in CDs for breaths and attacks with oxygen level were examined using LMEs models with either CD for breaths or attacks as the dependent variable, oxygen concentration as the explanatory variable and group as a random effect. For all models, model selection proceeded using maximum likelihood estimation, dropping variables one by one, starting with the variables with smallest t values. Variables were kept in the model if their removal resulted in significantly larger Akaike information criterion (AIC) value as indicated by likelihood ratio tests. The assumptions of homoscedasticity and normality of residuals were examined by visual inspection of residual‐fit plots.

Significance testing was employed to provide some indication of the strength of evidence for observed patterns, along with model *r* ^2^ values. This included marginal *r* ^2^ ($r_{m}^{2}$) and conditional *r* ^2^ ($r_{c}^{2}$) which indicate the variance explained by fixed factors and by both fixed and random factors, respectively (Nakagawa and Schielzeth, 2013). Across‐context repeatability of individual air‐breathing, aggression (number of attacks) and activity were calculated as adjusted (consistency) repeatability according to Nakagawa and Schielzeth (Nakagawa & Schielzeth, 2010), using variances calculated with LMEs that included fish ID and group as random effects. Oxygen concentration was included as a fixed effect in these calculations to account for mean changes in behaviours across treatment levels. Repeatability for fish tested in isolation vs. in a group, in normoxia and severe hypoxia (20% air saturation) was also calculated as adjusted repeatability using variances calculated with LMEs that included fish ID as a random effect. Again, as appropriate, either oxygen concentration or social treatment was included as fixed effects to account for mean changes in air‐breathing across treatment levels. *p*‐values are generally imprecise in model outputs and are arbitrary when used as thresholds for declaring statistical significance and problematic and limiting in several ways (Boos & Stefanski, 2011; Halsey, Curran‐Everett, Vowler, & Drummond, 2015). Thus, for all models, we treat *p*‐values as a continuous measure providing an approximate level of evidence against the null hypothesis (Fisher, 1956).

## 3 RESULTS

### 3.1 Air‐breathing alone vs. in a group

**Figure 1:**
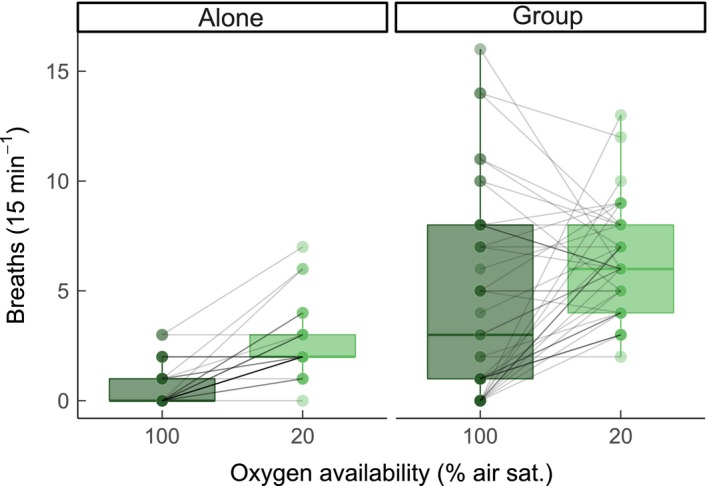
The frequency of air‐breathing performed by individual *Clarias gariepinus* when in isolation and when in a group context (group of four fish). Within each social treatment, the frequency of air‐breathing during exposure to aquatic normoxia (100% air saturation) and severe hypoxia (20% air saturation) is shown. Each data point represents one individual, with lines joining points for the same individual at each level of oxygen availability within each social treatment. Some points for individuals are overlapping; darker points within a social treatment (e.g. alone vs. group) represent more individuals at that value. Overall, there were 44 individuals (11 groups of four fish each). Boxplot lower and upper hinges represent the 25th and 75th percentiles, respectively; the horizontal line within the box represents the median; the length of whiskers represents the range data points between each hinge and 1.5× the difference between the 25th and 75th percentiles. Data beyond these limits are outliers

At any level of oxygen availability, fish took more air breaths when in groups as compared to when they were alone (Figure 1; LME, effect of social treatment, *F*_1, 126.09_ = 86.28, *p* < .0001). The large influence of social environment on individual air‐breathing was highlighted by the observation that fish in groups in normoxia took, on average, 8.2‐fold more air breaths than solitary fish exposed to severe hypoxia—a condition that is normally expected to stimulate air‐breathing. Indeed, air breathing increased in hypoxia in both isolation and in groups (Figure 1; LME, effect of oxygen, *F*_1, 115.89_ = 20.37, *p* < .0001). While estimated repeatability across conditions of normoxia and 20% air saturation appeared relatively high when fish were tested alone, there was a large degree of uncertainty and so the reliability of this estimate is questionable (*R* = 0.229, 95% CI: 0–0.56, *p* = .07). Repeatability of air‐breathing across social contexts (i.e. alone vs. in a group) was very low when fish were tested in normoxia (*R* = 0.037, 95% CI: 0–0.353, *p* = .430) or 20% air saturation (*R* = 0.128, 95% CI: 0–0.367, *p* = .180).

### 3.2 Individual variation in air‐breathing

When tested in groups, air‐breathing and attacks among individuals increased as aquatic oxygen availability declined (Table 1). The activity performed by individuals, in terms of total distance moved, was not directly affected by water oxygen availability but was strongly influenced by the number of attacks they performed (Table 1). Individuals within groups showed high repeatability across levels of oxygen availability for air‐breathing (*R* = 0.182, 95% CI: 0.043–0.294, *p* < .0001), attacks (*R* = 0.572, 95% CI: 0.416–0.687, *p* < .0001) and activity (*R* = 0.233, 95% CI: 0.104–0.354, *p* <.0001).

**Table 1:** Results of linear mixed effects models examining the factors influencing air‐breathing frequency, activity level and aggression in individual fish. Visualizations of key relationships among traits are shown in Figure 2

	Estimate	*SE*	*df*	*t*	*p*	$r_{M}^{2}$	$r_{C}^{2}$
Air breaths						0.440	0.596
Intercept	−1.458	5.088	35.62	−0.287	.776		
SMR	−1.282	0.888	26.04	−1.444	.161		
Mass	−1.840	2.747	31.27	−0.670	.508		
%MO_2 AIR_	0.021	0.016	34.44	1.311	.198		
Activity	2.095	0.257	195.43	8.138	<.0001		
Attacks	1.687	0.323	59.12	5.226	<.0001		
Oxygen availability							
20%	0.759	0.440	165.58	1.722	.087		
40%	0.2247	0.439	165.48	0.511	.610		
60%	−0.844	0.434	161.70	−1.946	.053		
80%	0.137	0.437	164.96	−0.314	.753		
Activity						0.152	0.204
Intercept	4.861	1.048	116.37	4.638	<.0001		
SMR	0.065	0.196	198.69	0.335	.738		
Mass	−0.927	0.587	137.89	−1.580	.116		
%MO_2 AIR_	0.001	0.003	100.25	0.444	.658		
Attacks	0.352	0.073	200.67	4.839	<.0001		
Oxygen availability							
20%	0.134	0.126	192.22	1.065	.288		
40%	0.116	0.126	192.19	0.921	.358		
60%	0.067	0.125	191.79	0.539	.591		
80%	−0.175	0.125	191.82	−1.405	.162		
Attacks						0.062	0.642
Intercept	−0.573	1.767	38.06	−0.324	.746		
SMR	−0.154	0.356	38.00	−0.432	.668		
Mass	0.466	1.007	38.00	0.462	.647		
%MO_2 AIR_	0.006	0.006	38.00	1.132	.264		
Oxygen availability							
20%	0.278	0.077	164.00	3.593	.0004		
40%	0.268	0.077	164.00	3.470	.0007		
60%	0.123	0.077	164.00	1.589	.114		
80%	0.140	0.077	164.00	1.802	.073		

SMR, standard metabolic rate.

**Figure 2:**
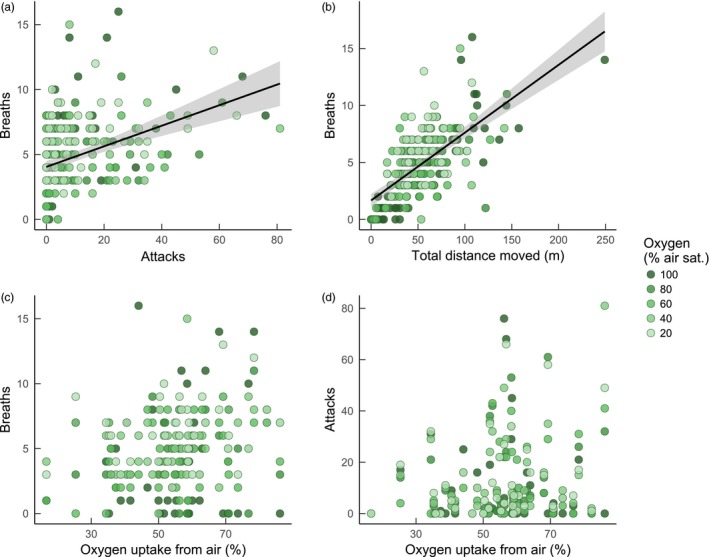
Interplay among air‐breathing frequency, aggression and oxygen uptake from air in *Clarias gariepinus* in social groups at various levels of water oxygen availability. Each data point is one individual fish within a group of four fish (11 groups in total). Panels a and b: relationships between either attack frequency (a) or activity (total distance moved; b) and air‐breathing frequency within a 15‐min period at a given levels of oxygen availability. Panels c and d: relationships between oxygen uptake from air while in respirometers (%MO_2_ _AIR_ in main text) and the frequency of air breaths (c) or attacks (d). Each point represents data for one individual fish within a group. Darker points represent data collected at higher levels of aquatic oxygen; each individual and group was tested at each oxygen level. Black lines represent linear regression lines through all individual data points for significant effects shown in Table 1; grey shaded area is the 95% confidence interval of the regression

Among individuals, those that were more aggressive (Table 1) and that were more active (Table 1) performed more air breaths when in groups (Figure 2a,b). Neither air‐breathing nor attacks were related to either SMR or %MO_2AIR_ among individuals in groups (Table 1; Figure 2c,d). When fish were tested for air‐breathing in isolation, individuals with a higher %MO_2AIR_ took more air breaths (LME, effect of %MO_2AIR_, *F*_1, 42.35_ = 6.256, *p* = .016). Air‐breathing was not related to SMR when fish were tested for air‐breathing in isolation.

**Figure 3:**
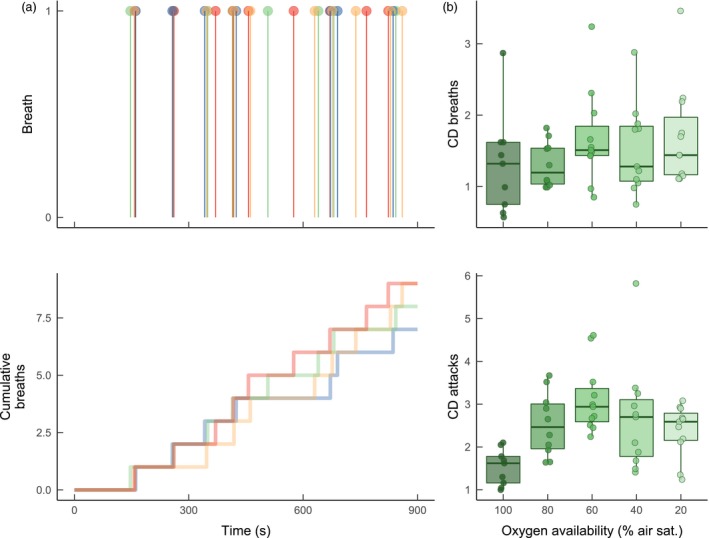
Temporal synchronization of air‐breathing and attacks in *Clarias gariepinus* in groups of four (*n* = 11 groups). Shown in a is an example plot of air‐breathing events for a particular group of fish (group 9 at an oxygen availability of 20% air saturation, CD for breaths = 1.749). The *x*‐axis shows the time throughout a 15‐min trial interval; each colour represents data for an individual fish within a group of 4. Top: vertical lines indicate occurrence of an air breath at that time. Bottom: cumulative count of breaths taken by each fish throughout the trial. Shown in B are the coefficients of dispersion (CD) for air breaths (top) and attacks (bottom). Each data point overlaid on the boxplots represents data for one group. All groups were tested at all levels of oxygen availability. Values for CD above 1.0 suggest temporal clustering of events, while values below 1.0 suggest more uniform temporal distributions. Boxplot lower and upper hinges represent the 25th and 75th percentiles, respectively; the horizontal line within the box represents the median; the length of whiskers represents the range data points between each hinge and 1.5× the difference between the 25th and 75th percentiles. Data beyond these limits are outliers

**Figure 4:**
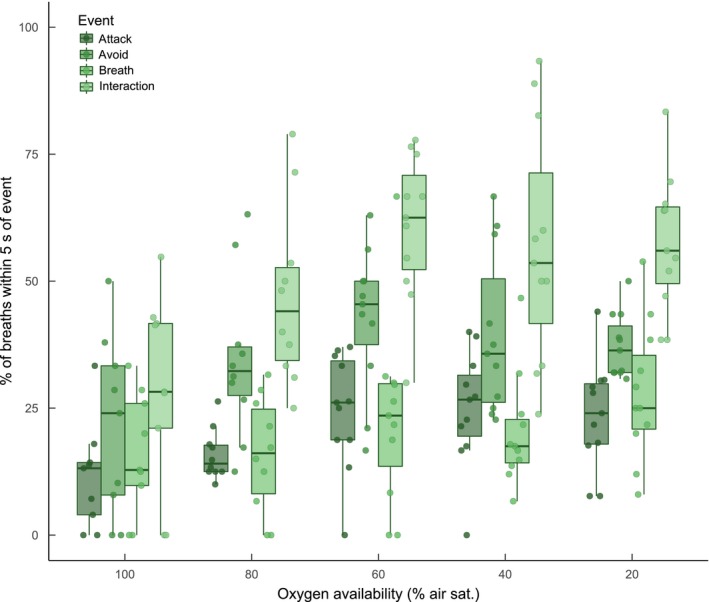
The relative frequency at which air‐breaths followed other behavioural events occurring within groups of *Clarias gariepinus*, at various levels of aquatic oxygen availability. The category “attack” refers to a breath being taken after that fish performed an attack; “avoid” refers to a breath after that fish performed an avoid; “interaction” includes attacks, pushes and avoids (see Section 2 for description of each). Each data point equals the mean for one group of four fish (*n* = 11 groups). All groups were measured at each level of oxygen availability. Boxplot lower and upper hinges represent the 25th and 75th percentiles, respectively; the horizontal line within the box represents the median; the length of whiskers represents the range data points between each hinge and 1.5× the difference between the 25th and 75th percentiles. Data beyond these limits are outliers

There was synchrony in both air‐breathing and attacks for fish observed in groups (C.D. for both was >1.0 across oxygen treatments, Figure 3). The degree of temporal synchrony for when fish engaged in attacks increased with hypoxia (LME, effect of oxygen availability, *F*_4, 37.57_ = 6.379, *p* = .001), while synchrony for air‐breathing remained relatively consistent across levels of oxygen availability. *c*. 15.9%–20.8% of air breaths occurred within 5 s after a breath being performed by another fish, with the exact percentage increasing with progressive hypoxia (Figure 4; GLM, effect of oxygen level, *F*_5,240_ = 9.40, *p* < .001). Similarly, 11.52%–24.9% of breaths occurred within 5 s of that fish attacking another fish and 21.3%–44.32% of breaths were performed within 5 s of that fish being attacked by another fish. Overall, 29.7%–63.27% of breaths by fish occurred within 5 s of that fish being involved in an agonistic interaction (either an attack, push or avoid), a significantly greater percentage than air‐breaths following breaths by another fish (Figure 4; GLM, effect of event type, *F*_3, 240_ = 50.422, *p* < .001).

### 3.3 Among‐group variation in air‐breathing

**Figure 5:**
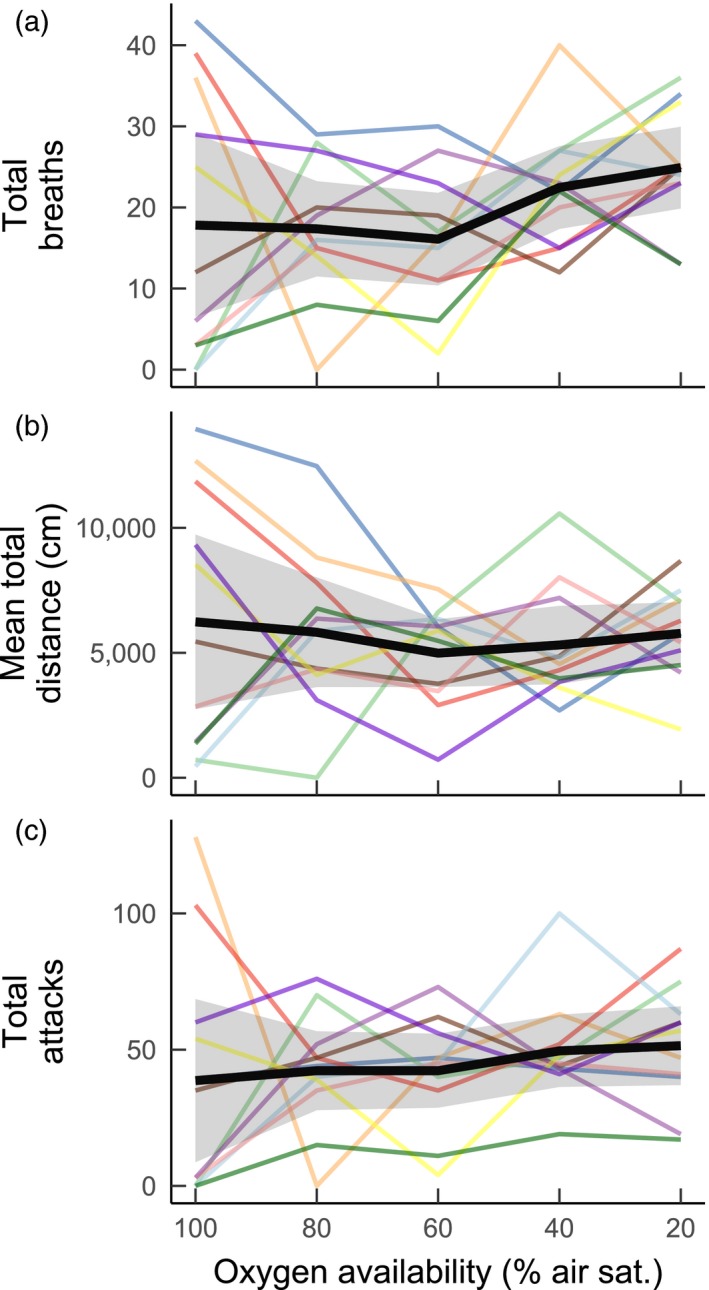
The effect of aquatic oxygen availability on: (a) air‐breathing; (b) activity (distance moved); and (c) attacks directed towards conspecifics within groups of *Clarias gariepinus*. Each thin line represent data for a different group of four fish (*n* = 11 groups). The heavy line represents the overall mean for all groups combined with 95% confidence intervals (grey shaded area)

The amount of air‐breathing, activity and attacks varied greatly among groups (Figure 5). For the number of attacks in particular, LME *r* ^2^_M_ was extremely low as compared to *r* ^2^_C_ (Table 1), suggesting a large amount of the variation in attacks was not attributable to model fixed effects but instead to variation among groups. The mean total air‐breaths performed by groups increased by approximately 40% as oxygen availability transitioned from 100% to 20% air saturation, but this group‐level increase in air‐breathing with hypoxia was not statistically significant (Figure 5a; LME, effect of oxygen, *F*_1, 44.15_ = 2.897, *p* = .0958). Group activity was not significantly affected by hypoxia (Figure 5b; LME, effects of oxygen, *F* = 0.2818, *p* = .8895), but the total number of attacks within groups increased with hypoxia (Figure 5c; LME, effect of hypoxia, *F*_1, 199.99_ = 2.676, *p* = .023). Overall, among‐group variation in behaviour tended to decrease as water oxygen availability declined (see 95% confidence intervals illustrated in Figure 5).

**Figure 6:**
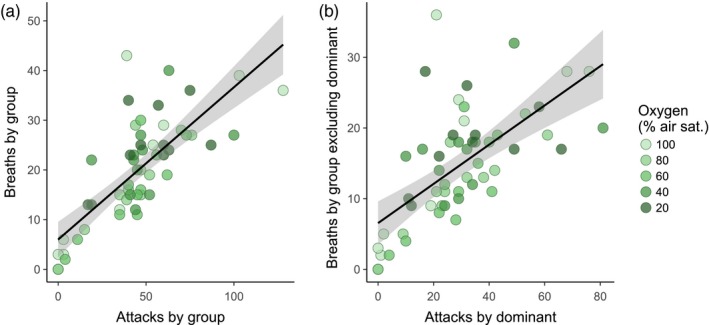
The effect of intraspecific aggression on air‐breathing in groups of *Clarias gariepinus*. Each data point represents data for one group of four fish (*n* = 11 groups). Panel a shows the relationship between the total attacks and total air breaths performed by all fish within each group. Panel b *only* shows the attacks performed by the most aggressive individual within each group (i.e. the dominant) and the breaths performed by *all other fish* but excluding the most aggressive individual. Darker points represent data collected at higher levels of aquatic oxygen; each individual and group was tested at each oxygen level. Black lines represent linear regression lines through all individual data points for significant effects shown in Table 2; grey shaded area is the 95% confidence interval of the regression

Groups with the most aggression, in terms of total number of attacks, also performed the most breaths (Table 2; Figure 6a). Groups that contained a particularly aggressive individual performed more air‐breathing overall, even after breaths by the dominant individual were excluded from group totals (Table 2; Figure 6b).

**Table 2:** Results of linear mixed effects models examining the factors influencing air breaths taken by groups of fish, with and without breaths taken by the dominant individual within each group. Visualizations of links among traits are shown in Figure 6

	Estimate	*SE*	*df*	*t*	*p*	$r_{M}^{2}$	$r_{C}^{2}$
Total breaths						0.635	0.773
Intercept	5.673	2.237	39.80	2.397	.021		
Attacks	0.314	0.031	45.14	10.112	<.0001		
Oxygen availability							
20%	3.062	2.309	39.23	1.326	.193		
40%	1.207	2.300	39.18	0.525	.603		
60%	−2.870	2.227	39.05	−1.260	.215		
80%	−1.598	2.227	39.05	−0.701	.487		
Total breaths excluding dominant						0.517	0.746
Intercept	7.119	2.056	31.97	3.464	<.0001		
Attacks by dominant	0.315	0.037	44.00	8.433	<.0001		
Oxygen availability							
20%	0.865	1.967	39.15	0.440	.662		
40%	−0.805	1.966	39.14	−0.409	.684		
60%	−4.108	1.939	39.00	−2.119	.041		
80%	−4.254	1.947	39.04	−2.184	.035		

## 4 DISCUSSION

These results demonstrate that variation in air‐breathing among groups of *C. gariepinus* is directly related to the amount of aggression occurring within social groups, with dominant individuals influencing the air‐breathing behaviour of the group as a whole. In a natural setting, this could translate into key individuals driving the degree of risk that other group members’ experience. These observations provide an extraordinary example whereby the influence of keystone individuals not only shapes the behaviour of groupmates (Modlmeier et al., 2014; Pruitt & Keiser, 2014), but seemingly supersedes the role of intrinsic physiological resource demand and environmental resource availability in determining individual behaviour. Specifically, although air‐breathing in fishes has a strong underlying physiological drive (Chapman & McKenzie, 2009; McKenzie et al., 1991, 2015) and is markedly stimulated when water oxygen availability falls, the influence of these factors can be obscured by behavioural interactions among individuals in a social environment.

Individuals breathed air far more often when in groups as compared to when they were in isolation, at both normoxia and severe hypoxia. There are at least two non‐mutually exclusive explanations for this observation. First, individual fish may be more likely to breathe air in the presence of conspecifics if being in a group makes it safer to come to the surface. Indeed, it has previously been shown that surfacing to air breathe can make individuals more prone to predation and synchronous air‐breathing may act to offset that risk (Chapman & Chapman, 1994; Kramer & Graham, 1976; Kramer et al., 1983). In the current study, air‐breathing was temporally clustered, providing evidence of synchrony and suggesting that an increase in the perceived safety of surfacing may have played a role in increasing the surfacing behaviour performed by individuals in groups. Another possibility is that individuals within groups actively cause others to breathe air. Previous reports of synchronous air‐breathing in fishes have implied a degree of intraspecific cooperation in surfacing behaviour (Chapman & Chapman, 1994), with groups of fish air‐breathing in a coordinated fashion in hypoxia to presumably reduce individual risk of predation. In contrast, we observed that the amount of air‐breathing performed by individuals in groups was clearly related to their level of activity and, specifically, the intensity of their agonistic interactions. Indeed, air breaths by an individual were more likely to follow an aggressive interaction than a breath by another fish. The current study examined air‐breathing in relatively small groups but, in larger groups, localized pockets of aggression between individuals followed by surfacing could prompt larger numbers of fish to surface in an apparently synchronous manner.

Given that air‐breathing is a chemoreflex stimulated by oxygen receptors (Chapman & McKenzie, 2009; McKenzie et al., 1991; Smatresk, 1988), the question is whether the surfacing reflected dynamic changes in oxygen demand caused by the aggressive interactions. Increased physical activity causes large increases in the intensity of surfacing in air‐breathing fishes that can be directly related to an increase in oxygen demand (Lefevre, Domenici, & McKenzie, 2014, 2016; McKenzie et al., 2012). Thus, increased activity during agonistic interactions may have caused individuals to take air breaths, including to recover from bursts of anaerobic swimming. It should be noted, however, that most aggressive interactions involved only quick bites or nudges, and even the most active catfish spent large amounts of time relatively motionless. Indeed, agonistic interactions were the main factor influencing the amount of activity displayed by individuals, with very little activity being performed outside of these events. Thus, although physical activity may have contributed to the increase in surfacing behaviour in groups, it seems unlikely to have been the sole factor. One possibility is that agonistic interactions cause an endocrine stress response, which may raise metabolic rate beyond the effects of the physical activity alone (Nadler, Killen, McClure, Munday, & McCormick, 2016; Sloman, Motherwell, O'Connor, & Taylor, 2000; Wendelaar Bonga, 1997). It is possible that this response is more pronounced in the subordinate individuals, thus providing a potential mechanism by which dominant individuals can cause others to surface first.

Additional research is therefore required to understand whether the links between air‐breathing and aggression within groups have a “physiological” basis, whereby effects on activity level and oxygen demand stimulated inescapable surfacing reflexes. If the responses are inescapable, then they may even be a cost of the adaptation in fishes, driving them to surface even when this is unnecessary or inappropriate. Interestingly, some air‐breathing fishes are capable of intense aerobic swimming while being denied access to aerial respiration but, if allowed access to air, will still surface even though it is seemingly unnecessary (Lefevre, Wang, Phuong, & Bayley, 2013; McKenzie et al., 2012). Although a different context from the current study, this suggests that physical activity (during swimming or during agonistic interactions) can trigger reflexive air‐breathing. This may be one reason why relatively few fish species are bimodal breathers (Lefevre, Domenici, et al., 2014). Although respiratory chemoreflexes in air‐breathing fishes transmit through the medulla oblongata in the brainstem (Taylor, Leite, McKenzie, & Wang, 2010), they have a large behavioural component, with surfacing influenced by higher order inputs such as perceived risk of predation (Chapman & McKenzie, 2009; McKenzie et al., 2015; Shingles et al., 2005). Thus, it is conceivable that once the physiological adaptations for air‐breathing are acquired, other drivers of this behaviour could evolve that are not immediately “physiological.”

The extreme difference in the frequency of air‐breathing observed between fish tested in isolation vs. in groups makes it difficult to evaluate the compromises made by individual fish to conform to the behaviour of the group, in terms of deviating from their apparent preferred frequency of air‐breathing in isolation. In groups, the fact that all fish performed air‐breathing much more than they would otherwise suggests that this was an emergent behaviour (Couzin & Krause, 2003; Marshall, Carter, Rowcliffe, & Cowlishaw, 2012) and an example of social facilitation of behaviour (Webster & Ward, 2011), seemingly precipitated by aggressive interactions. Furthermore, the aggressive individuals heavily influenced the air‐breathing behaviour of individuals within their social group, which is difficult to explain from the perspective of resource acquisition, and was not related to their individual basal oxygen demand. Indeed, the social influences drove air‐breathing to the extent of superseding any direct physiological drive stemming from exposure to environmental hypoxia. This illustrates how social context can strongly affect the behaviour of individuals and obscure any underlying links between intrinsic physiological or behavioural traits and the tendency to take risks (Herbert‐Read et al., 2013; Jolles, Taylor, & Manica, 2016; Killen, Marras, Metcalfe, McKenzie, & Domenici, 2013; Webster & Ward, 2011). The effects of social environment are typically ignored in studies of comparative physiology and sometimes even behavioural ecology, with animals examined in isolation. The study of air‐breathing in fishes is a prime example, having almost exclusively been studied from a physiological viewpoint (Graham, 1997, 2006) with relatively little attempt to understand costs and benefits of air‐breathing within the context of social interactions and predator avoidance (but see Chapman & Chapman, 1994; Kramer, 1987; Kramer & Graham, 1976; Kramer et al., 1983). This is despite the fact that air‐breathing is a form of resource acquisition that is in many ways analogous to foraging behaviour. Additional work is required to understand the ecological relevance of keystone individuals within the context of synchronous air‐breathing and group behaviours in general (Modlmeier et al., 2014; Pruitt & Keiser, 2014). For example, it would be useful to study the extent to which group phenotypic composition (in terms of physiological or behavioural traits)—as determined by passive and active among‐group assortment (Killen, Marras, Nadler, & Domenici, 2017)—may modulate the degree of influence that keystone individuals can have on the behaviour displayed by groupmates.

In conclusion, the amount of air‐breathing varied greatly among groups of *C. gariepinus* and the risk‐taking in the group was strongly tied to the amount of aggression performed by the most dominant and aggressive individual within each. The amount of aggression performed by individuals was positively correlated to their tendency to rely on aerial respiration when alone. Although hypoxia increased air‐breathing of isolated fish this physiological drive was obscured when they were in groups, because activity and agonistic interactions had such a dominant effect on air‐breathing even when oxygen availability was high. This demonstrates how social context can strongly affect the behaviour of individuals and obscure any underlying links between intrinsic physiology and tendency to take risks. Overall, these findings indicate that social environments should be taken into account when trying to determine the ecological relevance of individual variation and covariation in physiological and behavioural traits.

## AUTHORS’ CONTRIBUTIONS

S.S.K., A.J.E., D.J.M. and F.T.R. conceived the study; S.S.K., A.J.E., D.J.M. and N.M. collected the data; S.S.K. analysed the data; S.S.K. wrote initial draft of the manuscript; all authors contributed to further manuscript development and gave final approval for publication.

## DATA ACCESSABILITY

Data are deposited in the Dryad Digital Repository https://doi.org/10.5061/dryad.2575t (Killen, Esbaugh, Martins, Rantin, & McKenzie, 2017).
